# The Use of Electronic Nose as Alternative Non-Destructive Technique to Discriminate Flavored and Unflavored Olive Oils

**DOI:** 10.3390/foods10112886

**Published:** 2021-11-22

**Authors:** Nuno Rodrigues, Kevin Silva, Ana C. A. Veloso, José Alberto Pereira, António M. Peres

**Affiliations:** 1 Centro de Investigação de Montanha (CIMO), Instituto Politécnico de Bragança, Campus Santa Apolónia, 5300-253 Bragança, Portugal; nunorodrigues@ipb.pt (N.R.); kevin.silva@ipb.pt (K.S.); jpereira@ipb.pt (J.A.P.); 2 Instituto Politécnico de Coimbra, ISEC, DEQB, Rua Pedro Nunes, Quinta da Nora, 3030-199 Coimbra, Portugal; anaveloso@isec.pt; 3 CEB—Centre of Biological Engineering, Campus de Gualtar, University of Minho, 4710-057 Braga, Portugal

**Keywords:** cinnamon, garlic, rosemary, flavored oils, quality, oxidative stability, quality control, electronic nose, chemometrics

## Abstract

Cv. Arbequina extra virgin olive oils (EVOO) were flavored with cinnamon, garlic, and rosemary and characterized. Although flavoring significantly affected the physicochemical quality parameters, all oils fulfilled the legal thresholds for EVOO classification. Flavoring increased (20 to 40%) the total phenolic contents, whereas oxidative stability was dependent on the flavoring agent (a slight increase for rosemary and a decrease for cinnamon and garlic). Flavoring also had a significant impact on the sensory profiles. Unflavored oils, cinnamon, and garlic flavored oils had a fruity-ripe sensation while rosemary flavored oils were fruity-green oils. Fruit-related sensations, perceived in unflavored oils, disappeared with flavoring. Flavoring decreased the sweetness, enhanced the bitterness, and did not influence the pungency of the oils. According to the EU regulations, flavored oils cannot be commercialized as EVOO. Thus, to guarantee the legal labelling requirement and to meet the expectations of the market-specific consumers for differentiated olive oils, a lab-made electronic nose was applied. The device successfully discriminated unflavored from flavored oils and identified the type of flavoring agent (90 ± 10% of correct classifications for the repeated K-fold cross-validation method). Thus, the electronic nose could be used as a practical non-destructive preliminary classification tool for recognizing olive oils’ flavoring practice.

## 1. Introduction

Olive oil is a key ingredient of the Mediterranean diet, being documented several health benefits related to its consumption [1,2,3,4]. Olive oil is also worldwide appreciated due to the distinctive and valued sensory characteristics [5]. However, olive oil is quite prone to oxidation and thus, its physicochemical-sensory quality may decrease during storage, being needed to ensure that, when consumed, it still retains the characteristics that make it such an appreciated and healthy food [6]. For example, cv. Arbequina, one of the most used cultivars for the production of olive oil, usually presents a low shelf life in comparison to the traditional cultivars, mainly related to its low phenolic content [2]. One strategy to enhance the oil’s shelf life, as well as to meet the preferences of new generations of consumers, is through flavoring [7], enriching the oils with flavoring agents, enhancing both organoleptic and antioxidant characteristics [8]. Despite being an ancient practice [9], in recent years the demand and interest by consumers of flavored oils has increased [10]. The new generations of consumers are highly than ever informed about foods, increasing the demands for high-quality products that present simultaneously recognized healthy effects with appreciated sensory characteristics and sensations [7]. These aspects are a great challenge for industries which need constant innovation to respond to the consumers’ demands. So, olive oil producers have increased recently the introduction of flavors, sometimes with non-traditional flavor agents, mainly in the section of gourmet oils. Various matrices have been used to flavor olive oils ranging from essential oils, spices, and aromatic herbs with a predominance of basil, pepper, garlic, and bay leaf [2,7,8,11,12], truffles [13] and apple, banana, lemon, and orange [7,8]. Most of these flavoring agents have been used since ancient times in food, pharmaceutical, cosmetics, and perfumery seasonings due to the presence of several biological activities, including antimicrobial and antioxidant properties [14,15]. Furthermore, the use of different flavoring agents helps to maintain the nutritional value of foods, improve preservation qualities and enrich the flavor and aroma of various foods packed with olive oils [16]. However, according to European Union regulations, flavored olive oil cannot be marketed as extra virgin olive oil (EVOO), since according to the definition of extra virgin olive oil, it must only be extracted from superior quality olives, and may not undergo any treatment other than normal processing [17]. To be marketed, flavored oil can only be sold as oil processed with vegetables, fruits, herbs, or spices, with specific nutritional-sensory properties [18] or flavored olive oils where the agents are used and their concentrations are described, such as for example “olive oil flavored with…” [19]. On the other hand, the flavoring of olive oils may comprise a fraudulent practice, aiming to mask the sensory perception of organoleptic defects that if perceived implies a decrease of the olive oil commercial value [12].

Therefore, it is of utmost relevance to be capable to identifying flavored olive oils as well as to clearly differentiate the used flavoring agent. For this reason, there is considerable interest in the development of alternative instrumental techniques (non-invasive and non-destructive) to allow more objective, faster, and less expensive assessments of the sensory quality of olive oil products [20]. Electronic noses (E-noses) are one of the most non-invasive and non-destructive tools used in food analysis [21]. Several authors used E-noses namely to authenticate virgin olive oils (VOOs) according to the varietal or geographic origin of the olives [22], to monitor online the volatile profile during malaxation [23], to assess the evolution of the oxidative state of olive oils [24,25], to evaluate the aroma of VOOs [26], to assess sensory defects according to trained tasters [24], to classify commercial EVOO according to the perceived fruitiness intensity [27], or to predict fruity aroma intensity and defect presence in VOO [28]. Regarding flavored olive oils analysis, Pacioni et al. [29] applied an E-nose to discriminate commercial oils with truffle flavor aiming to detect possible frauds in the marketing of this product. Thus, this work aims to use a lab-made E-nose as a non-destructive, fast, and reliable tool to discriminate cv. Arbequina oils flavored with different flavoring agents (rosemary, cinnamon, and garlic) as well as to distinguish them from non-flavored olive oils.

## 2. Materials and Methods

### 2.1. Experimental Design

A batch of five litters from cv. Arbequina olive oil was collected from a local producer in the Macedo de Cavaleiros region (Northeast of Portugal), after a 12 month-period of storage, under the usual industrial conditions (dark and ambient temperature). This batch was divided into different 120-mL dark bottles. Three different flavoring agents were studied (cinnamon powder, garlic powder, and dried rosemary) and for each one, eight independent bottles were used, being also used the same number of bottles for the control (without any added flavoring agent). The flavoring agents, suitable for food usage, were from a Portuguese commercial brand, acquired in a local market and used as purchased. Flavoring was performed at a level of 1.5% *w*/*v* and was promoted by contact (maceration) for a period of 15 days, being the bottles stored, in dark conditions and at room temperature. After aromatization, the olive oils were filtered with Whatman No. 4 filter paper into 100 mL amber hermetic bottles, which were identified according to the type of aromatization. In summary, for each flavoring agent and control (unflavored), eight independent bottles were obtained and then analyzed. All oils were evaluated in duplicate.

### 2.2. Quality Parameters of Unflavored and Flavored Olive Oils

The quality parameters of the different olive oils were evaluated by determining free acidity (FA), peroxide value (PV), and specific extinction coefficients at 232 nm and 268 nm (K_232_ and K_268_) according to the standard methodologies described in the regulation of EC [30]. These parameters allow evaluating the acidity of the olive oil (% oleic acid), as well as the primary and secondary oxidation products. The descriptive profile of olive oil was evaluated by a sensory panel, composed by the panel head and eight trained tasters, who evaluated all olive oil samples, following the above mentioned European Community Regulation. The descriptive profile was evaluated through a test form according to the recommendations of the International Olive Council (IOC) [31] with some modifications described by Rodrigues et al. [5]. The olfactory intensities were graded using a continuous scale ranging from 0 (no perceived sensation) to 10 (maximum perceived intensity), being evaluated the intensity of fruitiness (ripe or green), fruit sensations, herbal sensations, and harmony. The intensities of the gustatory-retronasal attributes were graded in a similar scale, being evaluated the intensity of fruity (ripe or green), sweet, bitter, pungent, fruity sensations, herbal sensations, and harmony. Finally, global sensory perceptions were graded on a similar continuum, with complexity and persistence sensations being determined.

### 2.3. Oxidative Stability of Unflavored and Flavored Olive Oils

The oxidative stability (OS) was established by measuring the oxidation induction time using a Rancimat 743 apparatus (Metrohm CH, Switzerland) following the methodology previously described by Rodrigues et al. [5].

### 2.4. Total Phenol Content (TPC) of Unflavored and Flavored Olive Oils

The micro methanol-water extraction, was done according to Pizarro et al. [32], where in a 2 cm^3^ Eppendorf tube, 1 cm^3^ of MeOH-H_2_O 80% (*v*/*v*) was added to 0.5 g of olive oil, stirred for 1 min in vortex at the maximum speed and was subsequently centrifuged (Minispin 5452 Eppendorf) for 5 min at 13,200 rpm. The supernatant was removed into a 5 cm^3^ volumetric flask. This procedure was repeated two more times, replacing 1 cm^3^ of MeOH-H_2_O 80% (*v*/*v*) in Eppendorf. The three extracts were collected in the same flask, which in the end was made up of ultrapure water. Each extraction was performed in triplicate. To determine the TPC, a solution was prepared with 1500 mm^3^ of water, 100 mm^3^ of phenolic extract, and 100 mm^3^ of reagent Folin-Ciocalteu, vortexed for 3 s and allowed to react for 3 min. Subsequently, 300 mm^3^ of 20% sodium carbonate (*w*/*v*) was added, vortexed for 3 s and allowed to react for 60 min in the dark and at room temperature (20–22 °C). This procedure was carried out in triplicate, in the extracts obtained in the MeOH-H_2_O 80% extraction. All were evaluated by VIS spectroscopy, on a UV-VIS/UV-1280 Shimadzu spectrophotometer and detected at 765 nm. The results were expressed in Gallic acid equivalent (mg GAE kg^−1^).

### 2.5. E-Nose Analysis

#### 2.5.1. Lab-Made Device

The E-nose used was a lab-made all-in-one olfactory multi-sensor device and was previously described by Teixeira et al. [27]. The device comprised a sampling and a sensors array thermostized units (at 28 and 35 °C, respectively). A diaphragm vacuum air pump (model SC3502PM, from SKOOCOM, China) was used to deliver the headspace gas phase to the detectors. For cleaning purposes (system and sensors) nitrogen (UN 1066, Linde 089 cyl 02/15) was used at a constant flow until a stabilized baseline was achieved. The E-nose comprised of nine commercial MOS (S1: TGS 2600 B00; S2: TGS 2602; S3: TGS 2610 C00; S4: TGS 2611 C00; S5: TGS 2610 D00; S6: TGS 2611 E00; S7: TGS 2612; S8: TGS 826 A00; and S9: TGS 823 C12N), in which electrical properties change due to the adsorption of the volatile compounds on the surface of the MOS sensors. The electrical resistances (in ohms, Ω) were recorded using an Agilent data acquisition unit (model 34970A), controlled by an Agilent BenchLink Data Logger software.

#### 2.5.2. Olive Oil Samples Conditioning and Analysis

The olive oils analysis involved 0.5 mL of each sample, which were placed into a glass vial (25 mL) and allowed to equilibrate inside the sampling chamber for 13 min at 28 °C (selected following the recommendations of the International Olive Council (IOC) for olive oils sensory analysis). Simultaneously, the E-nose setup was cleaned for 13 min using a nitrogen flow to ensure reaching a stable signal baseline, indicative of a cleaned environment. Afterwards, the sample’s gas headspace was pumped into the detection chamber to interact with the MOS sensors for 2.5 min, being the resistance signals recorded at each 4 s.

#### 2.5.3. Data Acquisition, Feature Extraction, and Signal Treatment

The signals delivered by the nine MOS sensors were acquired by a data logger, being recorded 37–38 resistance values from each sensor and for each sample analyzed. To establish a representative and unique E-nose fingerprint of the volatile fraction of each olive oil, six feature extraction methods were evaluated [27,33], including the last response point (LP), the integral of the response curve (INT), the maximum response point (MAX), the minimum response point (MIN), the sum of the response curve (SUM) and the mean of the response curve (MEAN).

### 2.6. Statistical Analysis

One-way ANOVA followed, when appropriate, by the Tukey’s post-hoc multi-comparison test was applied to infer about the statistical significance of the olive oils flavoring practice on the physicochemical-sensory characteristics of cv. Arbequina EVOOs. The hierarchical clustering heatmap was applied to verify if the sensory profiles (olfactory or gustatory sensations) could be used as a tool for recognizing the type of olive oil (unflavored or flavored oils) as well as the flavoring agent (cinnamon, garlic or rosemary). The false color image with dendograms is obtained by computing the distance (dissimilarity) between both rows (olive oils) and columns (olfactory or gustatory sensations), using the Euclidean distance for computation. Linear discriminant analysis (LDA) was used as a supervised multivariate technique to evaluate the capability of the lab-made E-nose-MOS device to discriminate the oils under study. In the present work, a database comprising 54 treated signals for each sample (9 MOS × 6 different feature extractions) was obtained [34]. To establish the best LDA classification models, the simulated annealing (SA) algorithm was implemented to select the best subsets of non-redundant signals (among the 54) that allowed the best classification performance for the leave-one-out cross-validation (LOO-CV) and the repeated K-fold-CV procedures. The quality of the results was assessed considering the sensitivity (i.e., the percentage of correct classified samples) and the specificity (i.e., the percentage of samples correctly classified as belonging to a group in relation to all samples correctly or not classified as belonging to that specific group), as well as through 2D plots of the first two discriminant functions (DF). The statistical analysis was performed using the Sub-select [35] and MASS [36] packages of the open-source statistical program R (RStudio version 1.2.5033), at a 5% significance level.

## 3. Results and Discussion

### 3.1. Impact of Olive Oil Flavoring with Different Agents on the Physicochemical, Oxidative Stability and Sensory Profiles of cv. Arbequina Extra Virgin Olive Oils

In general, the effects of the flavoring of cv. Arbequina oils, with two typical agents (garlic and rosemary) and a less common one (cinnamon), on their physicochemical-sensory profiles were in line with the literature data [2,7,11,12,37,38,39,40]. As shown in Table 1, flavoring did not have a significant effect on FA, as also described by other researchers [7,37], but contributed to the reduction of the primary oxidation, as can be inferred by the significant decrease of the PV, mainly for rosemary and garlic flavored oils (a decrease of 26% and 11% compared with the unflavored oil, respectively), in line with the findings of Gambacorta et al. [38] and Sacchi et al. [39], when using several flavoring agents (rosemary, pepper, oregano, garlic or lemon). A similar decreasing trend was observed for *K*_232_, although in this case more expressive for garlic and cinnamon flavored oils (reduction of 29% and 24%, respectively). However, it should be noticed that cinnamon seemed to promote the formation of secondary oxidation by-products, as can be inferred by the increase of the *K*_268_ values, which even exceeded the legal maximum established by the Regulation of the EU Commission [30] for virgin olive oils classification. This negative effect was already observed by other researchers when olive oils were flavored with fruits, spices, or aromatic herbs [2,11,40].

From Table 1 it can be inferred that only the rosemary flavoring agent significantly increased the OS of the control oils (a rise of 14% compared to the control oils). A similar positive effect on the OS due to the addition of aromatic herbs or natural condiments has also been described in the literature for some flavoring agents [7,37,38]. Concerning the phenolics, it was found that the addition of flavoring agents increased the TPC by 20 to 40%, being expected an enhancement of the biological activity of these flavored oils, namely of their antioxidant activities as referred by Abenoza and Sánchez-Gimeno [37], for olive oils flavored with garlic and rosemary. The positive effects due to the flavoring process can be attributed to the migration of specific compounds from the aromatic plants to the olive oil [8,41].

The effects of flavoring on the sensory profiles of cv. Arbequina oils, was assessed based on the descriptive profiles established according to the recommendations of the IOC [31]. The flavoring procedure did not lead to the appearance of any sensory defect, being the sensations of cinnamon, garlic and rosemary easily perceived by the panelists in the corresponding flavored oils, with intensities of 6.8, 4.7, and 5.0, respectively. The olfactory and gustatory positive sensations detected and the intensities, according to an unstructured scale ranging from 0 to 10, are given in Table 2. Contrary to the control oils, cinnamon and garlic flavored oils, which showed intense fruity-ripely olfactory and gustatory sensations, rosemary flavored oils exhibited fruity-greenly sensations and thus, an enhanced freshness and improved properties. The fruit and dry herbs sensations, perceived in the unflavored oils, were clearly masked by the three studied flavoring agents, showing that the aromatization reduced the variety of the perceived sensory sensations. Bobiano et al. [12] also found that the addition of essential oils masked all positive and negative sensory attributes of the olive oils evaluated. In the present study, it was also observed that flavoring with rosemary led to the appearance of some herbaceous gustatory sensations, namely eucalyptus and pine attributes. In which concerns the basic tastes, all flavoring agents enhanced the bitter sensation and a decreased the sweet sensation, being the pungency not significantly influenced. Finally, the flavoring procedure promoted the harmony of the oils but decreased the complexity, not affecting the oils’ persistence. Globally, the sensory trends due to flavoring are, generally, in agreement with those reported by Abenoza and Sánchez-Gimeno [37], for oils flavored with garlic and rosemary. Among the three flavoring agents studied, rosemary showed the best potential to enhance the overall physicochemical and sensory quality of cv. Arbequina oils as well as their resistance against oxidation.

**Figure 1:**
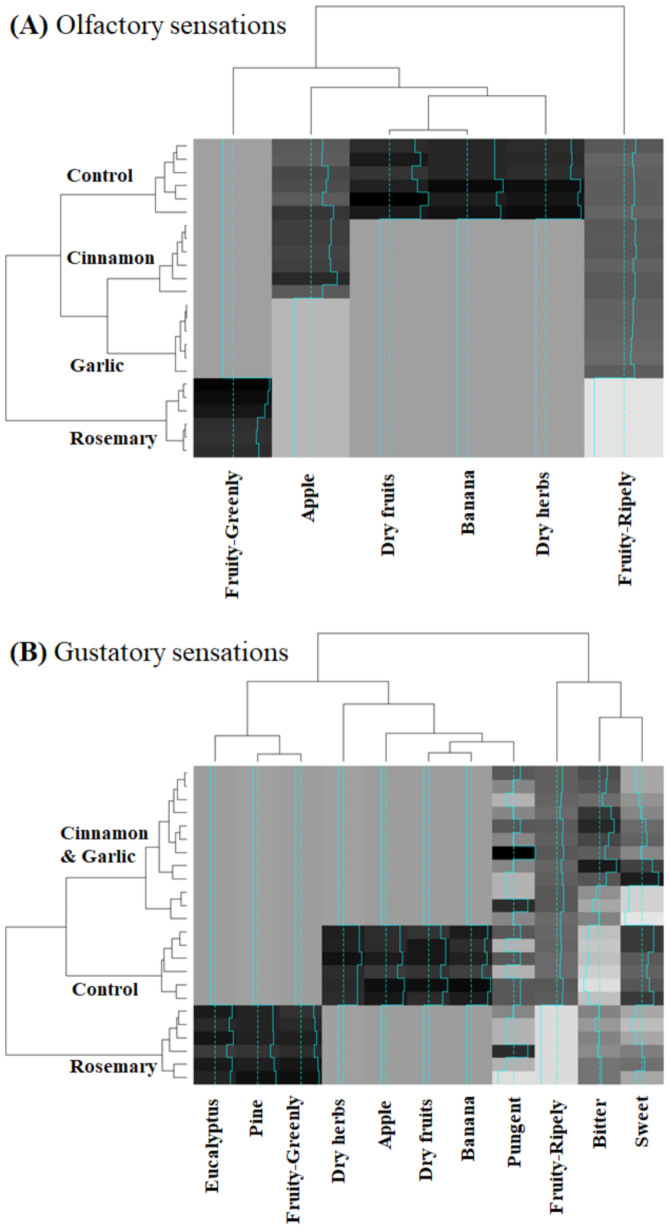
Hierarchical clustering heatmap (using Euclidean distances and Ward method) and respective dendograms for variables and unflavored cv. Arbequina oils (control oils) or oils flavored with cinnamon, garlic, or rosemary (white-black color scale: variable with lowest to highest influence, respectively).

Finally, as can be inferred from the hierarchical clustering heatmap and the respective dendograms, the olfactory (Figure 1A) and gustatory (Figure 1B) profiles established by the sensory panel, in diversity of sensations as well as in the perceived intensities, allow clustering each type of olive oil studied, especially for the olfactory sensations. As can be visualized (Figure 1), the olfactory profile possesses a superior clustering performance, being more perceived the flavoring impact on the olfactory sensations of the studied olive oils. In fact, the olfactory data (which are clustered into three groups: fruity-greenly, fruit plus herbaceous and fruity-ripely sensations) allowed the formation of four clusters (at a third level of the hierarchical tree), each corresponding to each type of oil. The gustatory sensations, at a third level of the hierarchical tree, only allowed the correct clustering of unflavored oils (control) and rosemary flavored oils, leading to a mix cluster for cinnamon and garlic flavored oils (Figure 1B). Indeed, as differentiation characteristics (Table 2 and Figure 1), unflavored cv. Arbequina oils (control oils) are mainly characterized by the fruit and dry herbs olfactory sensations; rosemary flavored oils by the fruity-greenly olfactory and gustatory sensations as well as by the eucalyptus and pine sensations; cinnamon flavored oils by the apple olfactory sensation; and garlic flavored oils by the overall lowest olfactory intensities.

However, the use of sensory data as a routine tool for classifying these oils, is a time-consuming and expensive procedure, taking into account the limited number of samples that can be evaluated per day, the scarcity of trained panelists, and the cost involved in the panelists’ training [12,20]. Thus, other analytical tools are required for routine fast and cost-effective analysis.

### 3.2. Performance of the Lab-Made E-Nose for Recognition of Unflavored and Flavored cv. Arbequina Extra Virgin Olive Oils

Regardless the positive effects of flavoring on some physicochemical and sensory attributes of olive oils, which have been reported and confirmed in the literature as well as in the present study, it should be remarked that, according to the EC Regulation [30], flavored olive oils cannot be labelled as EVOO. In fact, the commercialization of these oils requires the indication of the type of flavoring agent used as well as its concentration [17,18]. Therefore, it is of utmost relevance to develop non-invasive/non-destructive analytical techniques that can contribute to ensure the label correctness of this type of olive oils, which are quite appreciated by consumers and a common practice in Mediterranean countries. Among these techniques, E-noses have emerged as versatile, non-destructive sensing tools, which require a small amount of oil to be tested (usually less than 1 mL) and do not require any sample pre-treatment. Indeed, these olfactory sensing devices allowed establishing aroma fingerprints of olive oils [27,28] as well as to discriminate oils flavored or not with truffles [29].

**Figure 2:**
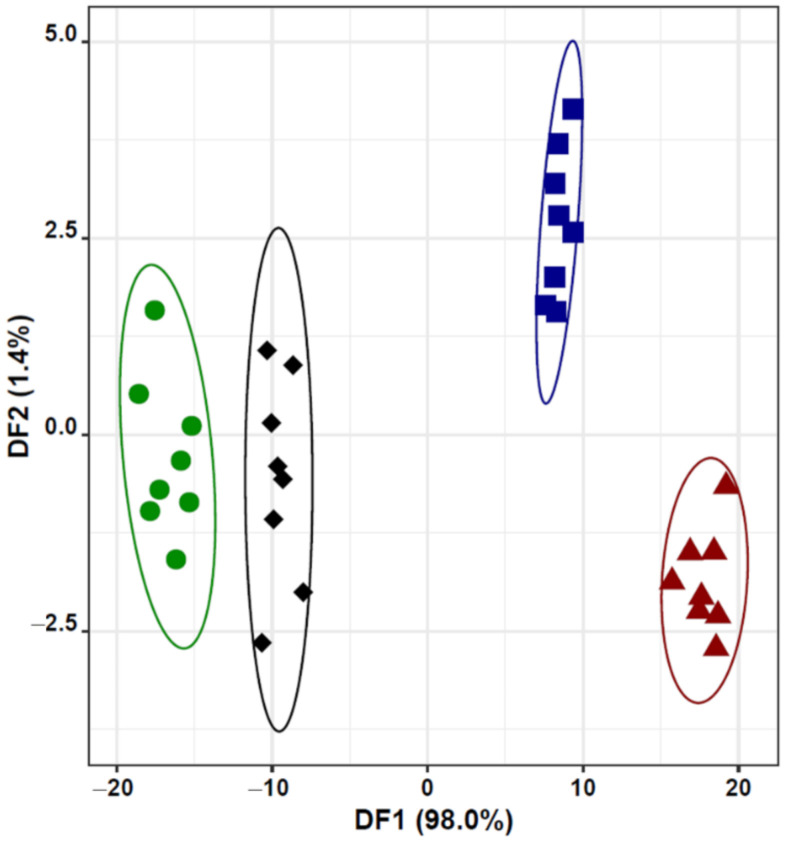
E-nose-MOS-LDA classification performance of unflavored and flavored cv. Arbequina olive oils: 2D plot of the first two discriminant functions (DF) based on 16 treated signals recorded by seven MOS (S5-LP, S6-LP, S1-INT, S2-INT, S6-INT, S5-MAX, S9-MAX, S2-MIN, S5-MIN, S8-MIN, S2-SUM, S5-SUM, S7-SUM, S8-SUM, S9-SUM, and S7-MEAN) selected by the SA algorithm: 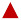 Unflavored oils (control); 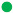 Cinnamon flavored oils; 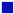 Garlic flavored oils; 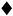 Rosemary flavored oils.

So, the capability of a lab-made E-nose was evaluated, with nine MOS sensors, to discriminate cv. Arbequina EVOOs from olive oils flavored with two traditional flavoring agents (garlic or rosemary) and one less commonly used (cinnamon) in the Mediterranean region. For this, the resistance signals recorded by the MOS sensors were treated according to six feature extraction techniques (LP, INT, MAX, MIN, SUM, and MEAN, totalizing 54 treated signals for each oil sample: 9 MOS × 6 feature extraction techniques). A classification model (E-nose-MOS-LDA-SA model) was established, based on 16 treated signals recorded by seven of the nine MOS sensors (i.e., S1, S2, S5, S6, S7, S8 and S9), which were selected by the SA algorithm. Two of the selected sensors (S1: TGS 2600 commercial sensor and S2: TGS 2602 commercial sensor) show specific reactivity toward some typical volatile organic compounds usually found in olive oils [42]. The first two DFs of the E-nose-MOS-LDA-SA model explained 99.4% of the data variability, allowing to correctly classify (100% of sensitivity and specificity; Table 3) all the unflavored and flavored olive oil samples under study (Figure 2).

It is interesting to note that, the groups’ differentiation achieved with the E-nose (Figure 2) and the sensory analysis, namely based on the intensities of the olfactory sensations perceived by the trained panelists (Figure 1A), is not similar. The E-nose discrimination capability followed the order “unflavored oils ≠ garlic flavored oils ≠ rosemary flavored oils ≠ cinnamon flavored oils”, instead the olfactory sensations perceived by the panelists resulted in a different order of differentiation, namely “unflavored oils ≠ cinnamon flavored oils ≠ garlic flavored oils ≠ rosemary flavored oils”. It should be kept in mind that although the E-nose aims to mimic the human olfactory capacity, the performance of the former is based on 8 MOS sensors and of the latter comprises hundreds of human olfactory cell receptors.

The E-nose-MOS-LDA-SA model also showed a satisfactory predictive performance, showing 94% and 95% of sensitivity and specificity for the LOO-CV variant (being the misclassification between cinnamon and rosemary flavored oils; Table 3). Furthermore, a mean sensitivity of 90 ± 10% was achieved for the repeated K-fold-CV variant (being no misclassification observed between unflavored and flavored oils). The E-nose power to discriminate the studied oils, namely unflavored from flavored oils, and among these latter ones, can be tentatively attributed to the overall differences between the olfactory profiles of the evaluated oils (fruity-ripely vs. fruity-greenly as well as presence vs. absence of fruity and herbaceous sensations). Indeed, the olfactory sensations and related intensities perceived by the sensory panel, allowed a clear differentiation of the four types of olive oils evaluated (unflavored and flavored oils), as shown in Figure 1. Lastly, the satisfactory classification results demonstrated the feasibility of using the E-nose as a non-destructive preliminary tool to discriminate cv. Arbequina oils flavored or not with cinnamon, garlic, or rosemary, allowing to identify flavored olive oils, which is legally required for commercialization.

## 4. Conclusions

Flavoring olive oils is a traditional practice in the Mediterranean region, usually resulting in olive oils with enhanced physicochemical and sensory characteristics. Among the three flavoring agents studied (cinnamon, garlic, and rosemary) it was found that, in general, rosemary-flavored oils showed the better overall quality and a higher oxidative stability. Since flavored oils cannot be commercialized as extra virgin olive oils, it was shown that a lab-made electronic nose, with metal oxide semiconductors sensors, could be used as a fast, simple, green, and non-destructive device for discriminating cv. Arbequina oils from the respective flavored oils, allowing to satisfactorily identifying the flavoring agent used. Thus, the future implementation of this type of electronic nose, coupled with chemometrics, as a flavoring sensor device for assessing the correctness of label of flavored oils, can be envisaged. These findings can contribute to enhancing the confidence of olive oil’s consumers when purchasing these differentiated and highly appreciated olive oils. In order to strengthen the study carried out, in the future, it would be interesting to assess the capability of the sensor device to detect different amounts of flavoring agents, aiming to establish the minimum value that could be detected. Furthermore, a broader number of flavoring agents should be evaluated, keeping in mind the need to carry out an external validation of the proposed approach.

## Figures and Tables

**Table 1:** Mean values (± standard deviation) of free acidity, peroxide value, extinction coefficients, oxidative stability and total phenols contents of unflavored (control) and flavored (cinnamon, garlic or rosemary) olive oils of cv. Arbequina olive oils (for each unflavored or flavored oil: *n* = 8).

Parameters ^1^	cv. Arbequina Olive Oils				*p*-Value ^2^
	Unflavored Oils (Control)	Flavored Oils			
		Cinnamon	Garlic	Rosemary	
FA (g oleic acid/100 g)	0.22 ± 0.00 ^A^	0.23 ± 0.01 ^A^	0.22 ± 0.01 ^A^	0.23 ± 0.00 ^A^	0.2942
PV (mEq O_2_/kg oil)	14.2 ± 0.8 ^A^	13.6 ± 0.4 ^A^	12.6 ± 0.3 ^B^	10.5 ± 0.4 ^C^	<0.0001
*K*_232_	2.37 ± 0.12 ^A^	1.80 ± 0.07 ^C^	1.69 ± 0.08 ^C^	2.11 ± 0.15 ^B^	<0.0001
*K*_268_	0.19 ± 0.01 ^B^	0.41 ± 0.02 ^A^	0.19 ± 0.02 ^B^	0.19 ± 0.01 ^B^	<0.0001
OS (h)	6.76 ± 0.38 ^B^	6.37 ± 0.14 ^B^	6.48 ± 0.18 ^B^	7.74 ± 0.57 ^A^	<0.0001
TPC (mg GAE/kg oil)	100 ± 3 ^C^	121 ± 14 ^B^	141 ± 5 ^A^	142 ± 7 ^A^	<0.0001

^1^ FA: free acidity; PV: peroxide value; K232 and K268: UV-Vis extinction coefficients at 232 and 268 nm, respectively; OS: oxidative stability; TPC: total phenols content. ^2^ *p*-values for the one-way ANOVA. Different letters in the same row show statistically differences (*p* < 0.05).

**Table 2:** Intensities of olfactory, gustatory and global sensations (mean ± standard deviation, for each unflavored or flavored oil: *n* = 8 olive oil bottles × 2 samples × 8 panelists) perceived by the trained sensory panelists.

Perceived Sensory Attributes		cv. Arbequina Olive Oils				*p*-Value ^1^
		Unflavored Oils (Control)	Flavored Oils			
			Cinnamon	Garlic	Rosemary	
Olfactory sensations						
Fruity	Greenly	0.0 ± 0.0 ^B^	0.0 ± 0.0 ^B^	0.0 ± 0.0 ^B^	3.8 ± 0.6 ^A^	<0.0001
	Ripely	7.2 ± 0.3 ^AB^	7.5 ± 0.2 ^A^	7.0 ± 0.2 ^B^	0.0 ± 0.0 ^C^	<0.0001
Fruit sensations	Apple	3.7 ± 0.5 ^A^	4.0 ± 0.5 ^A^	0.0 ± 0.0 ^B^	0.0 ± 0.0 ^B^	<0.0001
	Banana	1.8 ± 0.2 ^A^	0.0 ± 0.0 ^B^	0.0 ± 0.0 ^B^	0.0 ± 0.0 ^B^	<0.0001
	Dry fruits	1.4 ± 0.2 ^A^	0.0 ± 0.0 ^B^	0.0 ± 0.0 ^B^	0.0 ± 0.0 ^B^	<0.0001
Herbaceous sensations	Dry herbs	5.4 ± 0.6 ^A^	0.0 ± 0.0 ^B^	0.0 ± 0.0 ^B^	0.0 ± 0.0 ^B^	<0.0001
Harmony		7.4 ± 0.4 ^C^	8.1 ± 0.6 ^AB^	7.7 ± 0.2 ^BC^	8.7 ± 0.4 ^A^	0.0003
Gustatory sensations						
Fruity	Greenly	0.0 ± 0.0 ^B^	0.0 ± 0.0 ^B^	0.0 ± 0.0 ^B^	4.8 ± 0.5 ^A^	<0.0001
	Ripely	6.8 ± 0.4 ^A^	7.0 ± 0.4 ^A^	7.1 ± 0.4 ^A^	0.0 ± 0.0 ^B^	<0.0001
Basic tastes	Bitter	1.7 ± 0.2 ^C^	3.5 ± 0.3 ^A^	2.8 ± 0.5 ^B^	2.6 ± 0.2 ^B^	<0.0001
	Pungent	0.9 ± 0.1 ^A^	1.0 ± 0.1 ^A^	0.9 ± 0.1 ^A^	0.8 ± 0.1 ^A^	0.5407
	Sweet	5.4 ± 0.3 ^A^	5.1 ± 0.6 ^A^	3.7 ± 0.5 ^B^	4.2 ± 0.4 ^B^	<0.0001
Fruit sensations	Apple	3.3 ± 0.4 ^A^	0.0 ± 0.0 ^B^	0.0 ± 0.0 ^B^	0.0 ± 0.0 ^B^	0.0099
	Banana	2.1 ± 0.3 ^A^	0.0 ± 0.0 ^B^	0.0 ± 0.0 ^B^	0.0 ± 0.0 ^B^	<0.0001
	Dry fruits	1.3 ± 0.2 ^A^	0.0 ± 0.0 ^B^	0.0 ± 0.0 ^B^	0.0 ± 0.0 ^B^	0.0076
Herbaceous sensations	Dry herbs	6.5 ± 0.5 ^A^	0.0 ± 0.0 ^B^	0.0 ± 0.0 ^B^	0.0 ± 0.0 ^B^	<0.0001
	Eucalyptus	0.0 ± 0.0 ^B^	0.0 ± 0.0 ^B^	0.0 ± 0.0 ^B^	2.2 ± 0.3 ^A^	<0.0001
	Pine	0.0 ± 0.0 ^B^	0.0 ± 0.0 ^B^	0.0 ± 0.0 ^B^	4.1 ± 0.4 ^A^	<0.0001
Harmony		7.5 ± 0.3 ^B^	7.7 ± 0.4 ^AB^	7.7 ± 0.4 ^AB^	8.2 ± 0.4 ^A^	0.0232
Global sensations						
Complexity		5.4 ± 0.2 ^A^	3.6 ± 0.3 ^B^	2.2 ± 0.2 ^D^	2.8 ± 0.4 ^C^	<0.0001
Persistence		7.1 ± 0.2 ^A^	7.0 ± 0.6 ^A^	6.9 ± 0.9 ^A^	7.3 ± 0.6 ^A^	0.6301

^1^*P*-values for the one-way ANOVA. Different letters in the same row show statistically differences (*p* < 0.05).

**Table 3:** Confusion matrix for the E-nose-MOS-LDA-SA model: training and LOO-CV (in parentheses) procedures.

Actual Group (cv. Arbequina Oils)	Predicted Group (cv. Arbequina Oils)				Total	Sensitivity (%)
	Unflavored Oils	Cinnamon Flavored Oils	Garlic Flavored Oils	Rosemary Flavored Oils		
Unflavored oils	8 (8)	0 (0)	0 (0)	0 (0)	8 (8)	100
Cinnamon flavored oils	0 (0)	8 (6)	0 (0)	0 (2)	8 (8)	75
Garlic flavored oils	0 (0)	0 (0)	8 (8)	0 (0)	8 (8)	100
Rosemary flavored oils	0 (0)	0 (0)	0 (0)	8 (8)	8 (8)	100
Total	8 (8)	8 (6)	8 (8)	8 (10)	32 (8)	94
Specificity (%)	100 (100)	100 (100)	100 (100)	100 (80)	100 (95)	

## Data Availability

The data presented in this study are available on request from the corresponding authors.
